# Effects of Pre-Experience of Social Exclusion on Hypothalamus-Pituitary-Adrenal Axis and Catecholaminergic Responsiveness to Public Speaking Stress

**DOI:** 10.1371/journal.pone.0060433

**Published:** 2013-04-03

**Authors:** Ulrike Weik, Yvonne Kuepper, Juergen Hennig, Renate Deinzer

**Affiliations:** 1 Institute of Medical Psychology, University of Giessen, Giessen, Germany; 2 Center for Psychobiology and Behavioral Medicine, University of Giessen, Department of Psychology, Giessen, Germany; Max Planck Institute of Psychiatry, Germany

## Abstract

**Backround:**

Being socially excluded is associated with a variety of psychological changes and with an increased risk of disease. Today, the immediate physiological consequences of being socially excluded are not well understood. In two recent studies employing a standardized exclusion paradigm (Cyberball) we found social exclusion in this virtual game did not alter cortisol secretion directly. However, exclusion pre-experience suppresses the normal cortisol response to public speaking stress in women. The present study aims to replicate our previous finding and further elucidate it by analyzing for the first time whether this alteration of cortisol-responsiveness is associated to ACTH and whether the catecholaminergic system is affected as well.

**Methods:**

Women were randomly assigned to Cyberball-induced exclusion (SE, n = 22) or inclusion (SI, n = 21), respectively. Immediately afterwards they were subjected to public speaking stress. Salivary cortisol, plasma ACTH, catecholamines and estradiol were assessed as were psychological distress and mood.

**Results:**

Cyberball exclusion led to a highly significant immediate increase in negative affect in excluded women. After public speaking negative affect in included women increased as well and groups no longer differed. We replicate our previous finding of cortisol non-responsiveness to public speaking stress after exclusion pre-experience and find this effect to be significantly correlated with ACTH alterations. No such effects are observed for catecholamines.

**Conclusions:**

We replicated our previous study result of a supressed cortisol stress response after a short exclusion experience via Cyberball, thereby underlining the profound effects of social exclusion on a subsequent cortisol stress response. This further demonstrates that these alterations are associated with ACTH. Lack of effects on catecholamines is discussed in view of the tend-and-befriend hypothesis but also from a methodological perspective.

## Introduction

Lack of social support and social exclusion are associated with adverse effects on mental and physical health. They are often found to be correlated with disease, e.g. [1]–[7]. Gender differences reported in this context indicate women are more vulnerable to social triggers of health disturbances than men [8], [9]. Psychological and physiological mechanisms mediating between the degree of social integration and health are only partially understood. This holds true particularly with regard to physiology. The HPA system could be a mediating candidate within this context. Studies on the effects of social support, on HPA axis responsiveness indicate that social support might reduce the salivary cortisol stress response [10]–[13]. Less is known about the effects of social exclusion on cortisol secretion.

In two recent studies we employed a standardized exclusion paradigm (i.e. Cyberball) to analyse the effects of social exclusion on cortisol secretion [14], [15]. Cyberball is a virtual ball tossing game representing a commonly used paradigm to experimentally induce social exclusion [16], [17]. When playing Cyberball participants believe they are playing with other participants (who, in fact, are computer generated). During the game, the degree of social inclusion (i.e. how often they receive the ball from the other participants) is manipulated: “included” participants receive the ball regularly throughout the game while “excluded” participants receive no further ball after the first throws. In the last decade, Williams and co-workers as well as other scientists have undertaken a number of experimental studies in order to analyze effects of this virtual exclusion paradigm. They proved consistently robust immediate effects on psychological parameters, irrespective of the specific Cyberball design employed [18]–[24]. In addition, fMRI studies show that Cyberball-exclusion is associated with enhanced activation within the limbic system and in brain structures related to physical pain like the anterior cingulate cortex, insula, hippocampus and different areas within the prefrontal cortex [25]–[29].

With respect to HPA activity we and others found no significant immediate effects of Cyberball-induced exclusion on cortisol secretion [15], [30], [31]. We wondered whether Cyberball exclusion while not directly affecting cortisol secretion would perhaps increase the cortisol response to a subsequent public speaking stressor. Surprisingly, however, the effect we observed pointed in an unexpected direction: While men's cortisol response to public speaking stress was not affected by Cyberball-exclusion, excluded women showed a suppressed cortisol response to public speaking as compared to included women [14]. To our knowledge this was the first study to show such an effect of experimentally induced social exclusion on the cortisol response to a subsequent public speaking stressor, a result which is even more surprising considering that public speaking stress is known to be a valid psychological stressor in the sense of HPA axis responses [32]. Furthermore, the public speaking stressor we employed had consistently produced cortisol responses in men and women in our previous studies [33], [34]. Thus, the aim of the present study is to replicate this finding of cortisol non-responsiveness to acute psychological stress among previously socially excluded women and to further elucidate it by analyzing additional endocrine parameters.

In our previous study we discussed our finding in the context of fundamental (and presumably evolutionary-based) differences between men and women with respect to their social needs and their responses to threat [9], [35]. It has been proposed that under some circumstances women do not respond in a “fight or flight” manner, but rather in a “tend-and-befriend”-way allowing them to establish and re-establish social support, helping them to protect themselves and their offspring. Experiencing social exclusion may amplify this “tend-and-befriend”-response to stress which may dampen HPA axis responsiveness [35], [36]. One might therefore assume that the cortisol (non-) responsiveness is processed at a higher HPA level. However, a recent review indicates that there are several conditions under which dissociations between ACTH and cortisol can be observed [37].In order to test for an association between cortisol and ACTH stress responsiveness after Cyberball we thus included ACTH measures in this study.

According to the tend-and-befriend hypothesis, not only HPA axis responses are expected to be altered but also the responsiveness of the sympatho-adrenal system [35]. For this reason, we wondered whether catecholamine responses to public speaking stress are altered by social exclusion pre-experience and assessed epinephrine and norepinephrine responses to public speaking.

Another endocrine candidate to be considered when trying to explain lowered cortisol stress responses is estradiol. It is known to at least partly mediate sex differences in HPA stress responsiveness. A variety of animal and some human studies show dampening effects of estradiol on cortisol secretion [38], [39]. We thus controlled for estradiol in the present study by including it as covariate.

In summary, the present study aimed to replicate and further elucidate our previous finding of a suppression of the salivary cortisol stress response in women after Cyberball exclusion. We wanted to establish whether the alterations in cortisol were associated with ACTH alterations and whether the sympatho-adrenal system was affected as well.

## Materials and Methods

### Ethic Statement

The study was approved by the Ethics Committee of the Medical Faculty of the University of Duesseldorf, Germany and was found to conform to the guidelines of the World Health Organization (Declaration of Helsinki). All participants provided informed, written consent.

### Participants

Participants were 43 healthy female students between 18 and 35 years, recruited by advertisement on the University campus. They received a small monetary compensation (€25) for participation. Exclusion criteria were: acute or chronic infections, acute allergy, diseases of the adrenal gland, regular use of any medication, besides oral contraceptives (see below), gravidity, and acute or past mental illness.

### Experimental conditions

#### Independent variable: Experimental variation of social exclusion

Experimental variation of social exclusion (ostracism) was achieved via the Cyberball paradigm [16], [17]. Our female participants were made to believe that they were connected to three other players (actually computer generated) of the same sex, whose photographs and names are displayed on the computer screen [14]. Players are asked to throw a ball per mouse-click to each of the others. Every player is free to decide who receives the ball next. The ball is thrown 60 times. Two conditions were run: social exclusion (SE: after having received the ball three times, the participant does not receive it any more) and social inclusion (SI; control condition: the participant receives an average of every fourth ball.)

#### Randomization and blinding

Subjects were stratified with respect to oral contraceptive intake and randomly assigned to the experimental conditions. An equal number of cards containing the respective condition were put in sealed opaque envelopes prior to the study. Envelopes were shuffled immediately prior to each experiment and a person not involved in data assessment and not in contact with participants drew an envelope and set the respective experimental settings for the Cyberball game. Experimenters in direct contact with the subjects were blind to experimental conditions until the end of the experiment, when subjects were debriefed. Experimenters for the stress session differed from all other sessions in the experiment (see procedure). To keep participants blind with respect to hypotheses they were told the purpose of the study would be to examine effects of mental visualization task performance. The instructions they received comprised the German translation of the cover story on the welcome page of the Cyberball game which appear at the beginning of the game [17].

### Dependent variables

#### Salivary cortisol response

In order to test for effects of Cyberball on free cortisol saliva samples were taken every 15 minutes by means of Salivettes® (Sarstedt, Rommelsdorf, Germany) throughout the experiment and stored at −20°C until analysis. Salivary cortisol is considered the most valid parameter of HPA activation in psychoendocrinological studies [40]. Salivary cortisol levels were determined by the use of commercial enzyme-immunoassays (ELISA; IBL International®, Hamburg, Germany). All analyses were performed in duplicate using a fully automated analyzer (NexGen Four, Adaltis, Freiburg, Germany), within the same lot to avoid high inter-assay variation. The intra-assay-variation for all samples (CV) was below 5% in 91.3% of samples and below 10% in the remaining samples. Alterations in cortisol concentrations throughout the experiment were assessed as primary endocrine outcome variable.

#### ACTH and catecholamines

For analyzing effects of Cyberball on the stress responsiveness of ACTH and catecholamines to public speaking an indwelling venous catheter was placed in the subdominant arm when participants arrived at the laboratory and was kept free by saline solution and a mandrin placed in the catheter in the time between two samplings. All blood samples were collected on ice in EDTA-coated tubes and plasma was separated immediately after collection by centrifugation at 1700×g at 4°C for 15 minutes. Plasma samples were stored at −80°C until assayed. Plasma levels of ACTH were determined by the use of commercial enzyme immunoassays (ELISA; IBL International®, Hamburg, Germany). All analyses were performed in duplicate using a fully automated analyzer (NexGenFour, Adaltis, Freiburg, Germany) within the same lot to avoid high inter-assay variation. The intra-assay variation (CV) for ACTH was below 5% in 74.7% of samples and below 10% in the remaining samples. Analysis of plasma catecholamines was carried out by IBL (IBL, Hamburg, Germany) using an enzyme immunoassay (CatCombi, IBL). The intra- and interassay CVs of this standard CatCombi Kit are below 7.5 and 13.6, respectively.

#### Psychological parameters

Subjective mood during the respective experimental sections was assessed via short versions of the Multidimensional Mood Questionnaire (MDBF) [41] and the Differential Affect Scale (DAS) [42] with internal consistencies of .74–.88 and .54–.80, respectively. The short version of the MDBF consists of 16 items assessing three factors: mood (good vs. bad mood), alertness (alertness vs. tiredness), and calmness (calmness vs. agitation). Three scales of the DAS assessing happiness, depression, and anger, each by three items, were used. The assessments took place at baseline, immediately after Cyberball and after public speaking, respectively. In the instructions participants were asked to rate the feelings they had during the preceding section.

We measured the threat to fundamental needs via a standard questionnaire according to Williams and colleagues, e. g. [22], [24] consisting of 12 items assessing the effect of Cyberball on four needs: belonging, self-esteem, control and meaningful existence. Participants answered these questions by ratings on a 5-point scale, with 1 = not at all and 5 = very much.

### Control variables

The degree of the participants' social support was assessed by means of a standardized German questionnaire for the assessment of social support (Fragebogen zur sozialen Unterstützung) with the subscales ‘perceived social support’ and ‘social strain’ [43]. Furthermore, we used the German version of the IPC scales (internal-external control) for the assessment of locus of control. The IPC scales consist of three subscales: internal control, external control, and fatalistic external control [44], [45]. The internal consistencies of the social support questionnaire range between Cronbach's α = 0.81 and 0.93 and of the IPC scales between Cronbach's α = 0.91 and 0.98).

Several studies indicate that the cortisol stress response is related to the menstrual cycle and the intake of oral contraceptives and estradiol is considered to mediate these effects [38], [39]. We thus controlled for interindividual differences in estradiol which was assessed in plasma by the use of a commercial enzyme immunoassay (ELISA; IBL International®, Hamburg, Germany). All analyses were performed in duplicate using a fully automated analyzer (NexGen Four, Adaltis, Freiburg, Germany) within the same lot to avoid high inter-assay variation. The intra-assay-variation (CV) for estradiol was below 5% in 88% of samples and below 10% in the remaining samples. Additionally participants were asked about oral contraceptive intake and menstrual cycle phase.

### Manipulation checks

To assess the effectiveness of the Cyberball manipulations, standardized interviews followed at the end of each experiment. Participants were asked to describe any feelings and ideas they had regarding the Cyberball game. Thirteen participants socially excluded and no participant included stated doubts about whether the other players were indeed real (for more information see Results section).

### Procedure

A first appointment took place one week prior to the experiments. At that time, all subjects underwent an anamnestic interview in order to check for inclusion/exclusion criteria. Participants were informed about the details of the experiment and gave written consent. Additionally, they filled in the psychometric questionnaires (see above) and were photographed for the Cyberball game.

**Figure 1:**
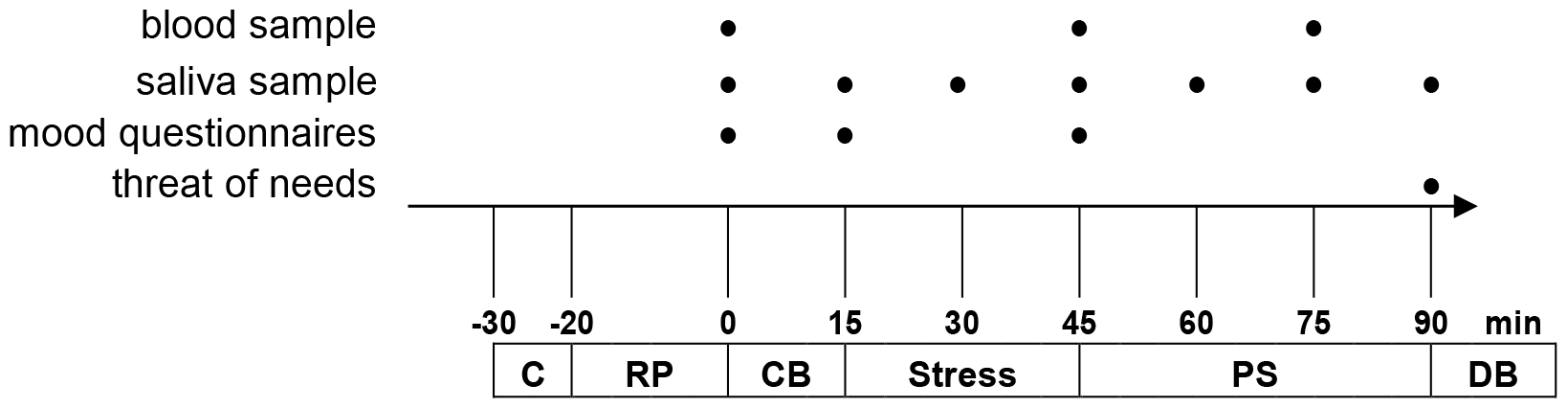
Assessment of dependent variables during the experiment. Assessment of blood and saliva samples, subjective mood and needs is indicated by a •. Abbreviations: C = placing the catheter; RP = rest period;CB = Cyberball; PS = poststress period; DB = debriefing.

Time points of assessment of the dependent variables during the experiment (second appointment) are shown in Figure 1. On experiment days, participants were asked to refrain from eating or drinking, except for water, 4 h prior to the beginning of the experiment. They were further asked not to drink coffee, tea, or caffeinated beverages, or to smoke for the same period of time. They also were instructed to avoid intense physical activities, sleep deprivation (less than 8 hours night sleep), and excessive alcohol consumption one day prior to and on experiment days.

All experiments started at 13:30 or 15:45 and groups were stratified with respect to time of beginning of the experiment. Experiments were subdivided into 4 sections: rest period, Cyberball, stress, poststress. During rest and poststress participants were provided with some comics to read.

After arriving at the laboratory, participants were seated in a quiet room, where the indwelling venous catheter was placed. Then the rest period (20 min) began.

Cyberball (15 min) began with placing the participant in a room equipped with a computer and giving them verbal instructions for the game (3–5 min). Afterwards, participants were provided with the same instructions on an information sheet and were asked to read them thoroughly in order to guarantee complete understanding of the (pretended) aim of the game (i.e. mental visualisation; see above). To leave the participant undisturbed while reading, the experimenter left the room for 4 min. Afterwards, the experimenter asked whether all instructions had been understood and answered questions by repeating the respective passages of the written instructions with the same or other words. The experimenter then told the participant that he had to leave the room in order to see whether the other participants were ready to go, too. A minute later the experimenter returned, reported that the others were ready, and pressed the start button of the game which lasted 4 min. Immediately after Cyberball another saliva sample was taken. The stress challenge (speech in front of a TV camera, i.e. public speaking) [14], [33], [34] took place in a separate room which was equipped with video cameras. They were connected to a supervisor room with a mixer desk and three video monitors visible to the participant when entering the room. The public speaking paradigm starts with an anticipation period (10 min) in which the participant is told to hold a speech in front of a TV camera and that more information will follow. After these 10 minutes the participant is informed of the topic of the speech (my positive and negative characteristics, what I think about them, how I judge them, and how they have influenced my life) and is informed of additional requirements to be fulfilled regarding duration and structure of the speech and the participant's expressive behavior during the speech. Now the participant has another 10 minutes to prepare the speech. After preparation the participant is asked to stand in front of the TV camera. The experimenter focuses the camera and then leaves the room. From now any further instructions are given from the supervisor room via microphone. The participant is now instructed to start the speech with her negative characteristics. After two minutes of talking the experimenter interrupts the speech regardless of the quality and reminds the participant of the requirements, repeats these requirements, and asks her to start again with the speech. After a total of 10 minutes after beginning the speech, the participant is informed that the 10-minute period is over and asked to sit down. The application of this stress paradigm in prior studies has led to a substantial impact on salivary cortisol secretion in men and women [14], [33], [34].

### Statistical analyses

In accordance with our study aims, we tested three main hypotheses:

a) Cyberball exclusion dampens the cortisol response to the subsequent public speaking stress, b) this cortisol response is associated with the ACTH response to public speaking and c) Cyberball exclusion affects catecholamine responses to subsequent public speaking. In order to control for interindividual differences in estradiol, baseline estradiol was included in all analyses as covariate according to its modulating role for stress responsiveness in women.

Prior to the analyses normal distribution assumption was tested by the Kolmogorov-Smirnov Goodness of Fit test for each parameter and each cell. Unless otherwise reported, all data were normally distributed (all p>0.05). Outlying data were excluded from the analyses on the basis of three standard deviations from the mean. Statistics were computed by use of SPSS 17. The intended level of significance was p≤0.05. Two-tailed p-values were computed.

To assess Cyberball effects on public speaking stress responses of salivary cortisol, epinephrine and norepinephrine in plasma, repeated measures ANCOVAs were computed, using the baseline values of the respective variable, baseline estradiol, and time of the experiment as covariates. Greenhouse-Geisser corrections were applied and original degrees of freedom together with Greenhouse-Geisser's ε are reported. In an explorative approach, we also analysed Cyberball effects on ACTH and estradiol in plasma, employing the same statistical procedures. The respective results are shown as differences to baseline, for absolute values see Tables S1 and S2 in the supplementary material.

In order to analyze whether alterations in salivary cortisol and ACTH were associated, we computed correlations of the differences between the respective measures taken immediately after public speaking stress and the corresponding baseline values. Exploratorily, we also computed correlations of cortisol alterations with catecholamines and estradiol responses.

The following analyses were run to test for psychological treatment effects:

To assess immediate Cyberball effects on psychological parameters, univariate ANCOVAs on the parameters assessed immediately after Cyberball were computed using the respective parameter at baseline, baseline estradiol, and time of the experiment as covariates.

Similarly, to assess Cyberball effects on the psychological response to public speaking stress, univariate ANCOVAs were run on the parameters assessed immediately after public speaking, using the respective parameter at baseline, baseline estradiol, and time of the experiment as covariates.

All ANCOVAs are presented with partial η^2^as measure of effect sizes.

## Results

Groups did not differ in any of the control variables (oral contraceptive intake, menstrual cycle phase, time of experiment, social support, and locus of control) or hormone baseline values except epinephrine (see Table 1). Blood parameters of one person in the exclusion group could not be analysed due to hemolysis. For one other person in the exclusion group and two persons in the inclusion group there was not enough plasma available to run catecholamine assays. Outlying values were found in the inclusion group only for the following parameters: salivary cortisol (2 cases), ACTH (2 cases), epinephrine (1 case), Differential Affect Scale Depression (1 case immediately after Cyberball) and Differential Affect Scale Anger (1 case immediately after Cyberball).

**Table 1:** Comparison of groups with respect to control variables and baseline values.

		Exclusion	Inclusion	X^2^	p
**Oral contraceptive intake**	yes/no	10/12	8/13	0.239	.760
**Menstrual cycle**	follicular/early luteal/late luteal/oral contraceptive	4/3/2/12	2/3/3/13	0.907	.923
**Time of experiment**	13:00/15:45	13/9	11/10	0.658	.763

		Mean	SD	Mean	SD	t	df*	p
**Locus of control**	fatalistic externality	23.23	(4.04)	24.43	(4.30)	0.95	(41)	0.35
	social externality	21.86	(3.60)	22.14	(4.94)	0.21	(41)	0.83
	internality	35.91	(3.08)	35.86	(2.46)	0.06	(41)	0.95
**Social support**	experienced social support	4.22	(0.36)	4.30	(0.44)	0.69	(41)	0.47
	social strain	1.80	(0.55)	1.94	(0.50)	0.87	(41)	0.39
**Endocrine parameters**	Salivary cortisol	8.16	(4.84)	7.84	(3.18)	0.24	(39)	0.81
	ACTH	11.36	(9.56)	9.14	(4.65)	0.92	(38)	0.36
	Epinephrine	60.02	(29.00)	38.91	(16.92)	2.70	(36)	0.01
	Norepinephrine	645.65	(322.47)	551.11	(274.75)	0.98	(37)	0.33
	Estradiol	47.19	(26.13)	54.85	(41.92)	−0.71	(40)	0.48
**MDBF**	Mood	84.67	(9.06)	83.01	(17.33)	0.40	(41)	0.69
	Alertness	62.06	(18.13)	65.56	(22.33)	−0.57	(41)	0.57
	Calmness	75.51	(14.98)	74.00	(20.23)	0.28	(41)	0.78
**DAS**	Happiness	49.45	(18.88)	58.05	(20.22)	−1.44	(41)	0.16
	Depression	7.92	(6.45)	8.33	(10.24)	−0.16	(40)	0.88
	Anger	5.12	(4.03)	4.15	(3.23)	0.86	(40)	0.40

DAS: Differential affect scale; MDBF: Multidimensional Mood Questionnaire;

* Differences in df due to outlying values and problems in parameter analyses (see first paragraph of Results).

### Psychological treatment effects

**Figure 2:**
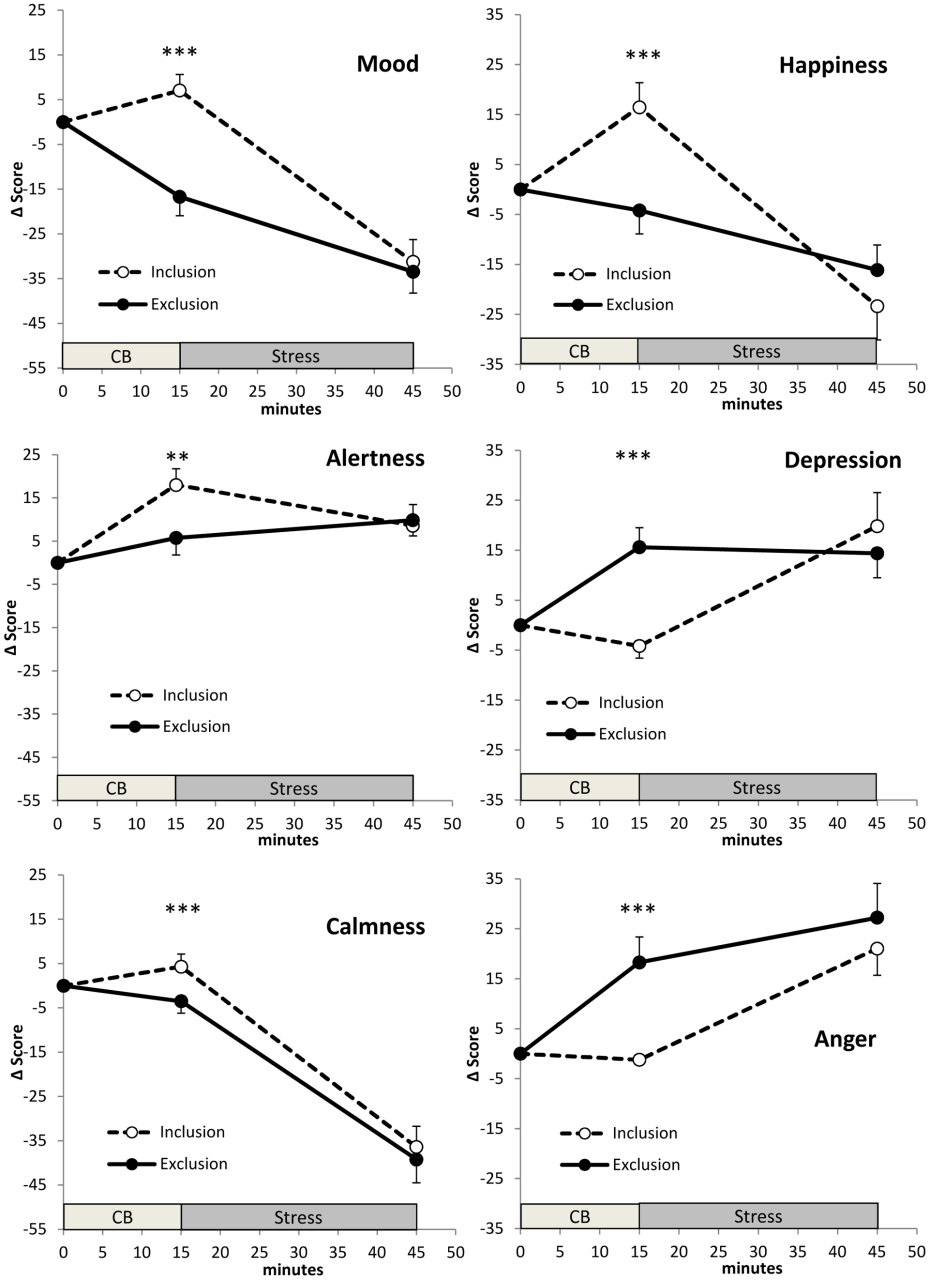
Mood Scales. Means and SEMs of differences from baseline (Δ = difference from baseline) of participants' mood ratings via Differential Affect Scales ‘happiness’, ‘depression’ and ‘anger’ and the scales ‘mood’, ‘alertness’ and ‘calmness’ of the Multidimensional Mood Questionaire. Groups differed significantly with respect to their feelings during Cyberball but not during public speaking stress (* = p<.05; ** = p<.01; *** = p<.001). For baseline values see Table 1, results of statistical analyses are shown in Table 3.

At the end of the experiment, groups differed highly significantly (all p<0.001) with respect to all needs measured (see Table 2). Furthermore, when rating the feelings they had during Cyberball, groups differed significantly with respect to all mood scales (all p<0.01). These differences vanished when they rated their feelings after public speaking stress (see Table 3 and Figure 2). Internal consistencies of the four needs, the DAS scales and the MDBF scales as observed in the present study are reported in Tables S3 and S4 in the supplemental material.

**Table 2:** Effects of Cyberball on threat of fundamental needs.

Threat of need	Exclusion (n = 21)		Inclusion (n = 21)				
	Mean	Stdev	Mean	Stdev	F(3/38)	η^2^	p
Belonging	3.95	.66	2.24	.73	64.87	.631	<.001
Control	4.03	.62	2.56	.60	59.42	.610	<.001
Self esteem	2.83	.89	1.67	.63	23.00	.377	<.001
Meaningful existence	3.33	.85	2.08	.61	28.16	.426	<.001

**Table 3:** Mood after Cyberball and after public speaking.

	after Cyberball			after public speaking		
	F(1/37)^3^	η^2^	p	F(1/37)	η^2^	p
Mood^1^	29.533	0.444	<.001	0.140	.004	.710
Alertness^1^	16.765	0.226	.003	0.129	.003	.722
Calmness^1^	8.090	0.183	.007	0.200	.005	.657
Happiness^2^	16.614	0.316	<.001	0.001	.000	.980
Depression^2^	23.163	0.398	<.001	0.745	.020	.394
Anger^2^	15.868	0.312	<.001	0.127	.004	.723

1 Multidimensional Mood Questionnaire;

2 Differential Affect Scale;

3 with baseline, baseline estradiol and time of experiment as covariates.

### Main Hypotheses

#### Cyberball effects on the stress response of salivary cortisol to public speaking

**Figure 3:**
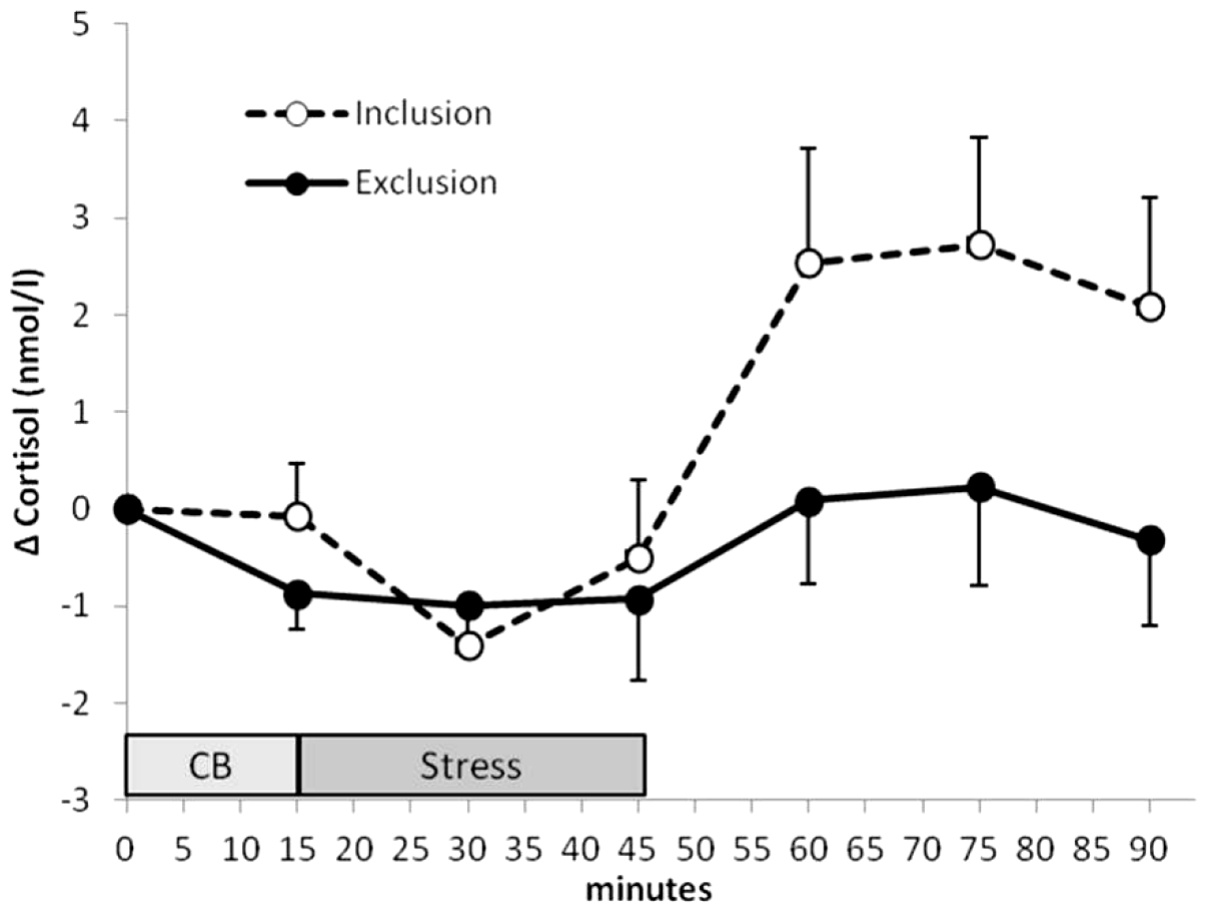
Salivary cortisol concentrations. Means and SEMs of differences from baseline of salivary cortisol concentrations (Δ = difference from baseline). Repeated Measures Analyses of Covariance reveal a significant Cyberball x time interaction (F = 3.771; p = .013). For baseline value see Table 1.

A significant Cyberball x time interaction was found for salivary cortisol (F(4/140) = 3.771, p = .013, η^2^ = .097, ε = .743; see Figure 3).

#### Correlation between cortisol and ACTH response to public speaking stress

A significant correlation was found between the cortisol and the ACTH response to stress both in the Cyberball exclusion and inclusion group (see Table 4).

**Table 4:** Correlations between stress-associated alterations in cortisol (difference immediately after public speaking minus baseline) and alterations in other endocrine parameters.

	Exclusion		Inclusion	
Pearson	*r*	*p*	*r*	*p*
ACTH	.567	.*008*	.426	.054
Epinephrine	.121	.610	−.112	.648
Norepinephrine	−.238	.312	.265	.272
Estradiol	.053	.818	−.131	.572

Spearman	*rho*	*p*	*rho*	*p*
ACTH	.629	.*002*	.475	.*030*
Epinephrine	.016	.948	.021	.932
Norepinephrine	−.150	.526	.235	.332
Estradiol	.118	.306	−.251	.274

#### Cyberball effects on stress responses of catecholamines to public speaking

**Figure 4:**
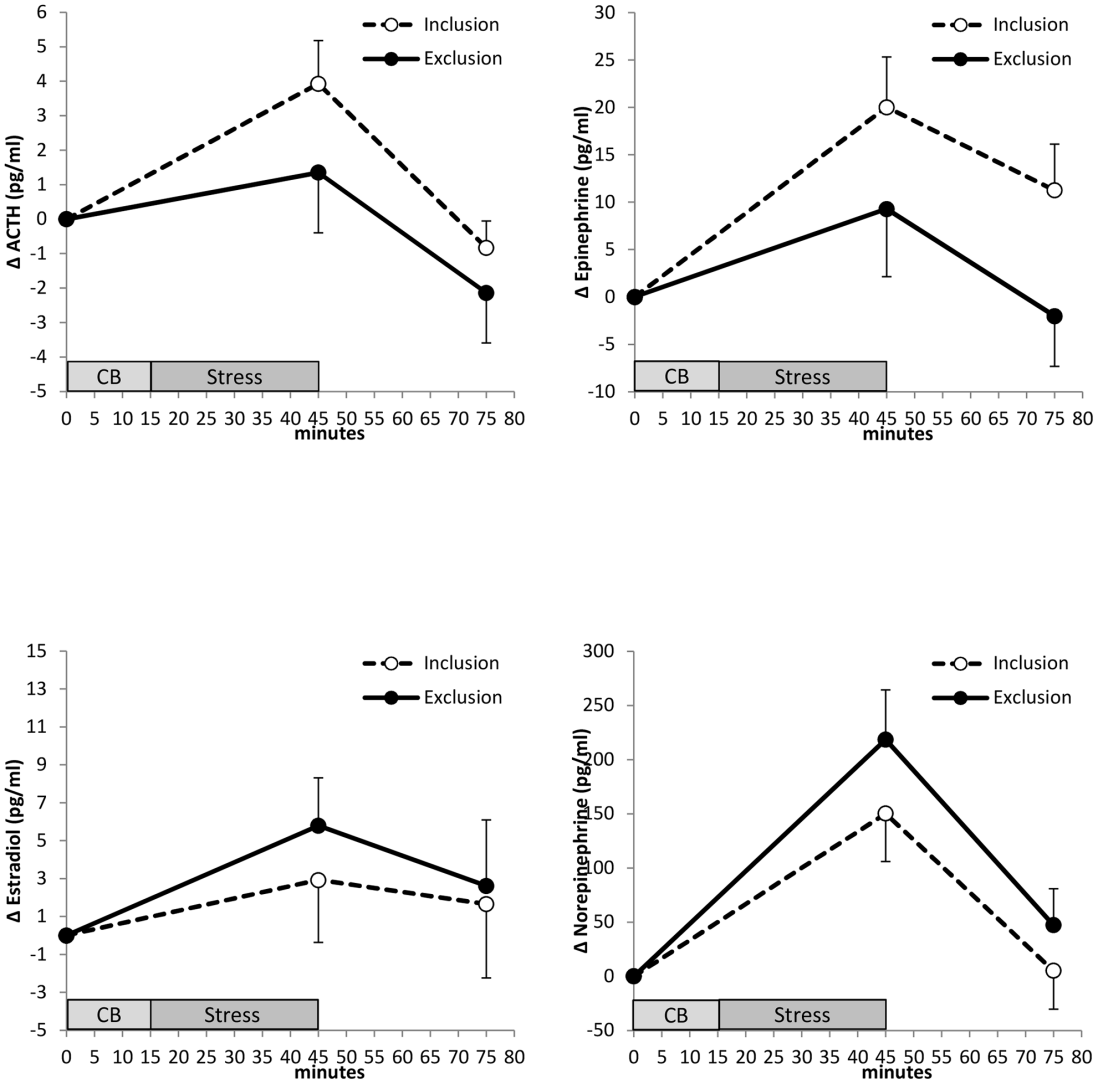
Plasma hormones. Means and SEMs of differences from baseline of plasma hormones (Δ = difference from baseline). For baseline values see Table 1. Repeated measures analyses of covariance reveal no significant effects for any of these parameters.

No significant main effect of Cyberball or Cyberball x time interaction was found for epinephrine (main effect: F(1/33) = .902, p = .349, η^2^ = .027; interaction: F(1/33) = .369, p = .548, η^2^ = .011) or norepinephrine (main effect: F(1/34) = 1.515, p = .227, η^2^ = .043; interaction: F(1/34) = .002, p = .961, η^2^<.001) (see Figure 4).

### Exploratory analyses

#### Effects of participants' doubts in the realness of the game on cortisol responsiveness

Since 13 women in the exclusion group reported at least some doubts as to the realness of the game, we assessed whether this affected cortisol responses. We thus compared cortisol responses of women in the exclusion group not reporting any doubts to those reporting doubts. Neither the main effect (F(1/17) = .468, p = .503, η^2^ = .027) nor the interaction with time (F(4/68) = 1.919, p = .156, η^2^ = .101, ε = .567) turned out to be significant.

#### Correlations between the cortisol and catecholamines responses to public speaking

No significant correlations were found between the stress response to public speaking of cortisol with the epinephrine or norepinephrine or estradiol response, respectively (see Table 4).

#### Cyberball effects on ACTH and estradiol

No significant main effect of Cyberball or Cyberball by time interaction was found for ACTH (p>.266, η^2^<0.036) or estradiol (p>.380, η^2^<0.020) (see Figure 4).

## Discussion

The present study investigated the effect of social exclusion via Cyberball on endocrine stress responses. One aim of the study was to replicate our previous result of a suppressed salivary cortisol response to public speaking stress in women pre-treated by this social exclusion paradigm [14]. Furthermore, this is the first study investigating whether the observed alterations in cortisol correspond with alterations in ACTH and whether there are effects of Cyberball on the responsiveness of catecholamines. With respect to salivary cortisol the present result corresponds to our previous finding, i.e. women failed to mount an acute cortisol stress response when being excluded immediately before stress. In order to better understand this cortisol non-responsiveness after Cyberball exclusion, we wondered whether ACTH was involved in this response. There are an increasing number of studies proving dissociations between these two parameters [37]. We thus aimed to test whether such a dissociation of cortisol and ACTH was observed here, too. In that case it would be difficult to argue that Cyberball effects on cortisol responsiveness were processed on higher levels of the HPA axis. Our data, however, show a strong association between ACTH and cortisol responsiveness after Cyberball exclusion and thereby support the assumption that the cortisol non-responsiveness is due to an overall change in HPA responsiveness.

Previously we discussed the observed female cortisol non-responsiveness to public speaking stress after Cyberball in the context of the tend-and-befriend hypothesis. This hypothesis postulates that survival of women and their offspring depends on functioning of social networks and their ability to (re-) establish them. According to that assumption, experiencing themselves as being excluded must reflect a tremendous threat for women (indeed, we found extremely high effect sizes for group differences with respect to all four fundamental needs). We therefore suggest that being excluded might be a major trigger for starting up the bio-behavioral tend-and-befriend program. Thus, another aim of the study was to analyze whether, besides the responsiveness of the HPA, the responsiveness of the other major stress system, i.e. the sympatho-adrenal system is also affected by this pre-treatment of social exclusion. Indeed, the tend-and-befriend hypothesis extends to the sympatho-adrenal system and dampened responsiveness is expected there as well. Our present data, however, are not in line with that expectation. We found no significant group differences in the catecholamine stress responses. With respect to epinephrine, visual inspection of Cyberball effects might indicate a dampened responsiveness of this parameter. The effect size is, however, too small to be statistically significant. Considering the fast temporal dynamics of the sympatho-adrenal system, the maximum Cyberball effects might have occurred at an earlier time point. In that case we would underestimate the real difference between groups. This might also explain the missing correlations between cortisol and catecholamine responsiveness. This uncovers a potential limitation of the blood sampling protocol. As we focused on the stress response to public speaking we assessed blood samples immediately before and after the stress protocol without interrupting the stress procedure by additional blood sampling.We could not thereby uncover short-term stress effects on the sympatho-adrenal system. In future research, alternative measures indicating autonomic response such as heart rate or galvanic skin response, which can be recorded continuously throughout the experiment, should thus be applied.

Nonetheless, the opposite direction of epinephrine and norepinephrine responses is worth to be considered in future studies as they might be related to functional differences of the two hormones reported earlier [46].

While we hitherto tried to explain our results by endocrine mechanisms, a closer analysis of the psychological alterations after public speaking stress might bring about an alternative explanationConsidering mood *changes* after Cyberball our data hint at a reduced psychological responsiveness to public speaking in excluded women. Such a finding would still correspond to the tend-and-befriend hypothesis, which also includes a dampening of strong psychological stress responses [36]. Another explanation for our psychological data could be that excluded women continued rumination about Cyberball during the public speaking paradigm and were thus not able to adequately respond to it. Future studies should consider this hypothesis and experimentally manipulate the degree of rumination after Cyberball exclusion.

Beyond this issue, several other open questions of equal importance remain. First of all, though Cyberball is designed to specifically manipulate feelings of social exclusion, it also alters affect *per se*. Thus it is not clear whether the effect we observe is specific to a social experience or would be observable after any induction of negative affect. According to the tend-and-befriend hypothesis, a social trigger of negative affect should lead to a greater group difference than a non-social trigger inducing a similar negative response in terms of mood. Thus, in a future study the effect of an alternative non-social manipulation of negative affect on the subsequent cortisol stress response should be analyzed. Another limitation of our study is the somewhat artificial way to experimentally manipulate social exclusion. One might doubt whether similar effects would be observable in more naturalistic settings (like e.g. the paradigm employed by Blackhart and colleagues) [47]. However, Cyberball exclusion not only strongly affects mood but also fundamental needs like that of belonging. It thus appears that despite its artificial nature it is a valid tool to induce feelings of being socially excluded. Nonetheless it would be interesting to directly compare the effect of this and more naturalistic designs.

Concluding, with this study, we replicated our previous finding by repeatedly demonstrating social exclusion via Cyberball to suppress a subsequent cortisol stress response in women. Additionally, we showed cortisol alterations here to be associated with ACTH. Moreover, as estradiol was included in our analyses as covariate, peripheral estradiol cannot readily explain that finding, neither intake of oral contraceptives nor menstrual cycle phase, because groups do not differ in these variables. Our data further suggest that the effects are confined to the HPA axis. The second endocrine stress system, i.e. the sympatho-adrenal system, was not affected in the same way by social exclusion pre-experience. With respect to the sympatho-adrenal system, however, as already discussed above, this result should be treated with caution for we cannot exclude possible effects arising at an earlier time point within the stress protocol. This should be analyzed in future work.

Finally, our results warrant further elucidation from theoretical, clinical, and methodological perspectives. From a theoretical perspective they pose questions on the mechanism by which the cortisol stress response is suppressed in such a quick and effective way after exclusion pre-experience. From a clinical perspective, our results indicate that social experiences may profoundly affect normal endocrine responses to a stress challenge. Even though the tend-and-befriend hypothesis suggests some benefits for women and their offspring when faced with immediate physical threats, these benefits might occur at the expense of physiological balance. Indeed, a dissociation of a strong psychological stress response and physiological non-responsiveness, as observed here, is considered to be an indicator of a deregulated system and of potential clinical harm [48]. From a methodological perspective, our experiment demonstrates that women's stress response is strongly affected by social pre-experiences. As we have shown previously, men are not affected in the same way [14]. It is thus mandatory to thoroughly control for such pre-experiences, particularly in research comparing cortisol responses of men and women to stress.

## Supporting Information

Table S1 Mean ± SD of salivary cortisol concentrations (nmol/l; sample 1 to 7) before, during and after public speaking in the exclusion and inclusion group respectively.
(DOCX)

Table S2 Mean ± SD of plasma endocrine parameter concentrations (pg/ml) before and after public speaking in the exclusion and inclusion group respectively.
(DOCX)

Table S3 Crohnbach's α for scales of the Multidimensional Mood Questionnaire (mood, alertness, calmness) and the scales of the Differential Affect Scale (happiness, depression, anger) after Cyberball and after public speaking.
(DOCX)

Table S4 Crohnbach's α for the four needs.
(DOCX)

## References

[1] Hawthorne G (2008) Perceived social isolation in a community sample: Its prevalence and correlates with aspects of peoples' life. Soc Psychiatry Psychiatr Epidemiol 43: 140–150. 17994175 10.1007/s00127-007-0279-8

[2] Torgrud LJ, Walker JR, Murray L, Cox BJ, Chartier M, et al (2004) Deficits in perceived social support associated with generalized social phobia. Cogn Behav Ther 33: 87–96. 15279315 10.1080/16506070410029577

[3] Montoya P, Larbig W, Braun C, Preissl H, Birbaumer N (2004) Influence of social support and emotional context on pain processing and magnetic brain reponses in fibromyalgia. Arthritis Rheum 50: 4035–4044. 15593181 10.1002/art.20660

[4] Prins JB, Huibers MJ, Servaes P, Van der Werf SP, Van der Meer JW, et al (2004) Social support and the persistence of complaints in chronic fatigue syndrome. Psychother Psychosom 73: 174–182. 15031590 10.1159/000076455

[5] Sorkin D, Rook KS, Lu JL (2002) Loneliness, lack of emotional support, lack of companionship, and the likelihood of having a heart condition in elderly patients. Ann Behav Med 24: 290–298. 12434940 10.1207/S15324796ABM2404_05

[6] Leserman J, Petitto JM, Golden RN, Gaynes BN, Gu H, et al (2000) Impact of stressful life events, depression, social support, coping and cortisol on progression to AIDS. Am J Psychiatry 157: 1221–1228. 10910783 10.1176/appi.ajp.157.8.1221

[7] Reynolds P, Kaplan GA (1990) Social connections and risk for cancer: Prospective evidence from the Alameda County Study. Behav Med 16: 101–110. 2224168 10.1080/08964289.1990.9934597

[8] Denton M, Prus S, Walters V (2004) Gender differences in health: A Canadian study of the psychosocial, structural and behavioral determinants of health. Soc Sci Med 58: 2585–2600. 15081207 10.1016/j.socscimed.2003.09.008

[9] Troisi A (2001) Gender differences in vulnerability to social stress. A Darwinian perspective. Physiol Behav 73: 443–449. 11438373 10.1016/s0031-9384(01)00459-0

[10] Heinrichs M, Baumgartner T, Kirschbaum C, Ehlert U (2003) Social support and oxytocin interact to suppress cortisol and subjective responses to psychosocial stress. Biol Psychiatry 54: 1389–1398. 14675803 10.1016/s0006-3223(03)00465-7

[11] Ditzen B, Neumann ID, Bodenmann G, von Dawans B, Turner RA, et al (2007) Effects of different kinds of couple interaction on cortisol and heart rate responses to stress in women. Psychoneuroendocrinology 32: 565–574. 17499441 10.1016/j.psyneuen.2007.03.011

[12] Ditzen B, Schmidt S, Strauss B, Nater UM, Ehlert U, et al (2008) Adult attachment and social support interact to reduce psychological but not cortisol responses to stress. J Psychosom Res 64: 479–486. 18440400 10.1016/j.jpsychores.2007.11.011

[13] Kirschbaum C, Klauer T, Fillip SH, Hellhammer DH (1995) Sex-specific effects of social support on cortisol and subjective responses to acute psychological stress. Psychosom Med 57: 23–31. 7732155 10.1097/00006842-199501000-00004

[14] Weik U, Maroof P, Zöller C, Deinzer R (2010) Pre-experience of social exclusion suppresses cortisol response to psychosocial stress in women but not in men. Horm Behav 58: 891–897. 20816966 10.1016/j.yhbeh.2010.08.018

[15] Zöller C, Maroof P, Weik U, Deinzer R (2010) No effects of social exclusion on salivary cortisol secretion in women in a randomized controlled study. Psychoneuroendocrinology 35: 1294–1298. 20334980 10.1016/j.psyneuen.2010.02.019

[16] Williams KD, Cheung CKT, Choi W (2000) Cyberostracism: Effects of being ignored over the internet. J Pers Soc Psychol 79: 748–762. 11079239 10.1037//0022-3514.79.5.748

[17] Williams KD, Jarvis B (2006) Cyberball: A program for use in research on interpersonal ostracism and acceptance. Behavior Research Methods 38: 174–180. 16817529 10.3758/bf03192765

[18] Alvares GA, Hickie IB, Guastella AJ (2010) Acute effects of intranasal oxytocin on subjective and behavioral responses to social rejection. Exp Clin Psychopharmacol 18: 316–21. 20695687 10.1037/a0019719

[19] Sebastian C, Viding E, Williams KD, Blakemore SJ (2010) Social brain development and the affective consequences of ostracism in adolescence. Brain Cogn 72: 134–45. 19628323 10.1016/j.bandc.2009.06.008

[20] Boyes ME, French DJ (2009) Having a cyberball: Using a ball-throwing game as an experimental social stressor to examine the relationship between neuroticism and coping. Personal and Individual Differences 47: 396–401.

[21] Lau G, Moulds ML, Richardson R (2009) Ostracism: How Much It Hurts Depends on How You Remember It. Emotion 9: 430–434. 19485620 10.1037/a0015350

[22] Van Beest I, Williams KD (2006) When inclusion costs and ostracism pays, ostracism still hurts. J Pers Soc Psychol 91: 918–928. 17059310 10.1037/0022-3514.91.5.918

[23] Williams KD, Zadro L (2005) Ostracism. The indiscriminate early detection system. In: KD Kipplin, JP Forgas, W von Hippel (Eds.). The social outcast. Psychology Press, New York, pp.19–34.

[24] Zadro L, Williams KD, Richardson R (2004) How low can you go? Ostracism by a computer is sufficient to lower self-reported levels of belonging, control, self-esteem, and meaningful existence. J Exp Soc Psychol 40: 560–567.

[25] Bolling DZ, Pitskel NB, Deen B, Crowley MJ, McPartland JC, et al (2011) Dissociable brain mechanisms for processing social exclusion and rule violation. NeuroImage 54: 2462–2471. 20974272 10.1016/j.neuroimage.2010.10.049 PMC3006641

[26] Masten CL, Eisenberger NI, Borofsky L, Pfeifer JH, McNealy K, et al (2009) Neural correlates of social exclusion during adolescence: Understanding the distress of peer rejection. Social Cognitive and Affective Neuroscience 4: 143–157. 19470528 10.1093/scan/nsp007 PMC2686232

[27] Onoda K, Okamoto Y, Nakashima K, Nottono H, Ura M, et al (2009) Decreased ventral anterior cingulate cortex activity is associated with reduced social pain during emotional support. Soc Neurosci 4: 443–54. 19562631 10.1080/17470910902955884

[28] Onoda K, Okamoto Y, Nakashima K, Nittono H, Yoshimura S, et al (2010) Does low self-esteem enhance social pain? The relationship between trait self-esteem and anterior cingulated cortex activation induced by ostracism. Soc Cogn Affect Neurosci 5: 385–391. 20144945 10.1093/scan/nsq002 PMC2999754

[29] Eisenberger NI, Lieberman MD, Williams KD (2003) Does rejection hurt? An fMRI study of social exclusion. Science 302: 290–292. 14551436 10.1126/science.1089134

[30] Geniole SN, Carréa JM, McCormick CM (2011) State, not trait, neuroendocrine function predicts costly reactive aggression in men after social exclusion and inclusion. Biological Psychology 87: 137–145. 21382439 10.1016/j.biopsycho.2011.02.020

[31] Zwolinski J (2012) Psychological and Neuroendocrine Reactivity to Ostracism. Aggress Behav doi:10.1002/ab.21411. 10.1002/ab.21411 22331583

[32] Dickerson SS, Kemeny ME (2004) Acute stressors and cortisol responses: a theoretical integration and synthesis of laboratory research. Psychol Bull 130: 355–91. 15122924 10.1037/0033-2909.130.3.355

[33] Deinzer R, Granrath N, Stuhl H, Twork L, Idel H, et al (2004) Acute stress effects on Il-1 β responses to pathogens in a human in vivo model. Brain Behav Immun 18: 458–467. 15265539 10.1016/j.bbi.2003.11.008

[34] Weik U, Herforth A, Kolb-Bachofen V, Deinzer R (2008) Acute stress induces proinflammatory signaling at chronic inflammation sites. Psychosom Med 70: 906–912. 18799429 10.1097/PSY.0b013e3181835bf3

[35] Taylor SE, Klein LC, Lewis BP, Gruenewald TL, Gurung RAR, et al (2000) Biobehavioral responses to stress in females: Tend-and-befriend, not fight-or-flight. Psychol Rev 107: 411–429. 10941275 10.1037/0033-295x.107.3.411

[36] Klein LC, Corwin EJ (2002) Seeing the unexpected: how sex differences in stress response may provide a new perspective on the manifestation of psychiatric illness. Current psychiatric reports 4: 441–448. 10.1007/s11920-002-0072-z 12441024

[37] Bornstein SR, Engeland WC, Ehrhart-Bornstein M, Herman JP (2008) Dissociation of ACTH and glucocorticoids. Trends Endocrinol Metab 19: 175–180. 18394919 10.1016/j.tem.2008.01.009

[38] Kajantie E, Phillips DI (2006) The effects of sex and hormonal status on the physiological response to acute psychosocial stress. Psychoneuroendocrinology 31: 151–78. 16139959 10.1016/j.psyneuen.2005.07.002

[39] Kudielka BM, Hellhammer DH, Wüst S (2009) Why do we respond so differently? Reviewing determinants of human salivary cortisol responses to challenge. Psychoneuroendocrinology 34: 2–18. 19041187 10.1016/j.psyneuen.2008.10.004

[40] Hellhammer DH, Wüst S, Kudielka BM (2009) Salivary cortisol as a biomarker in stress research. Psychoneuroendocrinology 34: 163–171. 19095358 10.1016/j.psyneuen.2008.10.026

[41] Steyer R, Schwenkmezger P, Notz P, Eid M (1997) Der Mehrdimensionale Befindlichkeitsfragebogen (MDBF). Göttingen: Hogrefe.

[42] Merten J, Krause R (1993) DAS (Differentielle Affekt Skala). Arbeiten der Fachrichtung Psychologie, Universität des Saarlandes, Saarbrücken.

[43] Fydrich T, Sommer G, Brähler E (2007) Fragebogen zur Sozialen Unterstützung (F-SozU). Manual, Göttingen: Hogrefe.

[44] Levenson H (1972) Distinctions within the concept of internal-external control: Development of a new scale. Proceedings of the 80th Annual Convention of the American Psychological Association 7: 261–262.

[45] Krampen G (1981) IPC-Fragebogen zur Kontrollüberzeugung. Göttingen: Hogrefe.

[46] Goldstein D (Sep 2010) Adrenaline and Noradrenaline. In: eLS. John Wiley & Sons Ltd, Chichester. http://www.els.net [doi: 10.1002/9780470015902.a0001401.pub2].

[47] Blackhart GC, Eckel LA, Tice DM (2007) Salivary cortisol in response to acute social rejection and acceptance by peers. Biol Psychol 75: 267–276. 17485157 10.1016/j.biopsycho.2007.03.005

[48] McEwen BS (1998) Protective and damaging effects of stress mediators. N Engl J Med 338: 171–179. 9428819 10.1056/NEJM199801153380307

