# Supplementary material for: Effects of Pre-Experience of Social Exclusion on Hypothalamus-Pituitary-Adrenal Axis and Catecholaminergic Responsiveness to Public Speaking Stress
Source: PLoS One. 2013 Apr 3;8(4):e60433. doi: 10.1371/journal.pone.0060433 (PMC3616100; doi:10.1371/journal.pone.0060433)
Supplement: Table S1 — Mean ± SD of salivary cortisol concentrations (nmol/l; sample 1 to 7) before, during and after public speaking in the exclusion and inclusion group respectively. (DOCX) [file pone.0060433.s001.docx]

**Table S1:** Mean ± SD of salivary cortisol concentrations (nmol/l; sample 1 to 7)

before, during and after public speaking in the exclusion and inclusion group

respectively.

|  | **Exclusion** | **Inclusion** |
| --- | --- | --- |
| baseline | 8.16 ± 4.8 | 7.84 ± 3.2 |
| before stress | 7.29 ± 4.5 | 7.76 ± 4.1 |
| during stress | 7.17 ± 4.4 | 6.44 ± 3.1 |
| immediately after stress | 7.23 ± 4.9 | 7.34 ± 3.3 |
| poststress 1 | 8.24 ± 4.6 | 10.38 ± 5.5 |
| poststress 2 | 8.38 ± 4.8 | 10.56 ± 5.2 |
| poststress 3 | 7.83 ± 4.4 | 9.92 ± 5.1 |
