# Supplementary material for: Effects of Pre-Experience of Social Exclusion on Hypothalamus-Pituitary-Adrenal Axis and Catecholaminergic Responsiveness to Public Speaking Stress
Source: PLoS One. 2013 Apr 3;8(4):e60433. doi: 10.1371/journal.pone.0060433 (PMC3616100; doi:10.1371/journal.pone.0060433)
Supplement: Table S2 — Mean ± SD of plasma endocrine parameter concentrations (pg/ml) before and after public speaking in the exclusion and inclusion group respectively. (DOCX) [file pone.0060433.s002.docx]

**Table S2:** Mean ± SD of plasma endocrine parameter concentrations (pg/ml)

before and after public speaking in the exclusion and inclusion group respectively.

|  |  | **Exclusion** | **Inclusion** |
| --- | --- | --- | --- |
| ACTH | baseline | 11.36 ± 9.5 | 9.14 ± 4.6 |
|  | immediately after stress | 12.71 ± 9.5 | 13.06 ± 6.4 |
|  | poststress | 9.22 ± 5.6 | 8.31 ± 3.2 |
|  |  |  |  |
| Epinephrine | baseline | 60.02 ± 29.0 | 38.91 ± 16.9 |
|  | immediately stress | 69.30 ± 39.6 | 58.93 ± 21.1 |
|  | poststress | 58.00 ± 33.5 | 50.18 ± 27.2 |
|  |  |  |  |
| Norepinephrine | baseline | 645.65 ± 322.5 | 551.11 ± 274.8 |
|  | immediately stress | 864.20 ± 437.9 | 701.32 ± 304.8 |
|  | poststress | 692.80 ± 337.9 | 556.21 ± 270.6 |
|  |  |  |  |
| Estradiol | baseline | 47.19 ± 26.1 | 54.85 ± 41.9 |
|  | immediately stress | 52.98 ± 29.3 | 57.78 ± 45.9 |
|  | poststress | 49.80 ± 30.4 | 56.51 ± 39.5 |
