# Supplementary material for: Effects of Pre-Experience of Social Exclusion on Hypothalamus-Pituitary-Adrenal Axis and Catecholaminergic Responsiveness to Public Speaking Stress
Source: PLoS One. 2013 Apr 3;8(4):e60433. doi: 10.1371/journal.pone.0060433 (PMC3616100; doi:10.1371/journal.pone.0060433)
Supplement: Table S3 — Crohnbach's α for scales of the Multidimensional Mood Questionnaire (mood, alertness, calmness) and the scales of the Differential Affect Scale (happiness, depression, anger) after Cyberball and after public speaking. (DOCX) [file pone.0060433.s003.docx]

**Table S3:** Crohnbach’s α for scales of the Multidimensional Mood

Questionnaire (mood, alertness, calmness) and the scales of the

Differential Affect Scale (happiness, depression, anger) after

Cyberball and after public speaking

|  | **after Cyberball** | **after public speaking** |
| --- | --- | --- |
| Mood | .78 | .69 |
| Alertness | .82 | .55 |
| Calmness | .46 | .82 |
| Happiness | .96 | .93 |
| Depression | .81 | .86 |
| Anger | .94 | .91 |
