# Supplementary material for: Effects of Pre-Experience of Social Exclusion on Hypothalamus-Pituitary-Adrenal Axis and Catecholaminergic Responsiveness to Public Speaking Stress
Source: PLoS One. 2013 Apr 3;8(4):e60433. doi: 10.1371/journal.pone.0060433 (PMC3616100; doi:10.1371/journal.pone.0060433)
Supplement: Table S4 — Crohnbach's α for the four needs. (DOCX) [file pone.0060433.s004.docx]

**Table S4:** Crohnbach’s α for the four needs

| Selfesteem | .67 |
| --- | --- |
| Belonging | .74 |
| Control | .57 |
| Meaningful existence | .58 |
